# Prediction of recurrent glioblastoma after laser interstitial thermal therapy: The role of diffusion imaging

**DOI:** 10.1093/noajnl/vdz021

**Published:** 2019-08-20

**Authors:** Abdelkader Mahammedi, Suha Bachir, Edward J Escott, Gene H Barnett, Alireza M Mohammadi, Mykol Larvie

**Affiliations:** 1 Department of Radiology, Cleveland Clinic, Cleveland, Ohio; 2 Department of Neurosurgery, Cleveland Clinic, Cleveland, Ohio; 3 Department of Neurosurgery, Cleveland Clinic Lerner College of Medicine of Case Western Reserve University, Cleveland, Ohio; 4 Department of Radiology, University of Kentucky, Lexington, Kentucky; 5 Department of Pediatrics and Human Genetics, Cincinnati Children’s Hospital Medical Center, Cincinnati, Ohio

**Keywords:** apparent diffusion coefficient, diffusion-weighted image, glioblastoma, laser interstitial thermal therapy, MRI-guided laser interstitial thermal therapy

## Abstract

**Background:**

Evaluate the utility of diffusion-weighted imaging (DWI) for the assessment of local recurrence of glioblastoma (GBM) on imaging performed 24 h following MRI-guided laser interstitial thermal therapy (LITT). We hypothesize that microscopic peritumoral infiltration correlates with early subtle variations on DWI images and apparent diffusion coefficient (ADC) maps.

**Methods:**

Of 64 patients with GBM treated with LITT, 39 had MRI scans within 24 h after undergoing LITT. Patterns on DWI images and ADC maps 24 h following LITT were correlated with areas of future GBM recurrence identified through coregistration of subsequent MRI examinations. In the areas of suspected recurrence within the periphery of post-LITT lesions, signal intensity values on ADC maps were recorded and compared with the remaining peritumoral ring.

**Results:**

Thirty-nine patients with GBM met the inclusion criteria. For predicting recurrent GBM, areas of decreased DWI signal and increased signal on ADC maps within the expected peritumoral ring of restricted diffusion identified 24 h following LITT showed 86.1% sensitivity, 75.2% specificity, and high correlation (*r* = 0.53) with future areas of GBM recurrence (*P* < .01). Areas of future recurrence demonstrated a 37% increase in the ADC value (*P* < .001), compared with findings in the surrounding treated peritumoral region. A significantly greater area under the receiver operating characteristics curve was determined for ADC values (*P* < .01).

**Conclusions:**

DWI obtained 24 h following LITT can help predict the location of GBM recurrence months before the development of abnormal enhancement. This may alter future treatment planning, perhaps suggesting areas that may be targeted for additional therapy.

**Key Points**

1. Diffusion-weighted imaging may predict the location of post-LITT GBM recurrence 24 h after LITT.

2. This can be detected months before the development of abnormal enhancement.

3. All post-LITT lesions have peripheral restricted diffusion 24 h after LITT.

**Importance of the Study**

- Laser interstitial thermal therapy (LITT) is an emerging minimally invasive therapeutic modality that is increasingly used in the treatment of diverse brain lesions such as brain tumors and epileptogenic foci. There is relatively little published data to inform optimal use of LITT. We present cases and data that demonstrate the role of apparent diffusion coefficient (ADC) maps in the prediction of recurrent GBM on MRI following LITT.

- The results of this study suggest that within the complex area of signal abnormality seen in patients with GBM following LITT, areas of decreased DWI signal and increased ADC along the expected peritumoral restricted diffusion on scans performed 24 h after LITT predict likely sites of tumor recurrence. This information might alter treatment planning, perhaps suggesting areas that may be targeted for additional therapy.

Laser interstitial thermal therapy (LITT) utilizes a small fiberoptic laser catheter to deliver heat precisely, which causes focal destruction of tissue.^1^ It was first described by Bown in 1983 using bare infrared fibers. Laser therapy is particularly suited to MRI because optical fibers are not affected by and do not affect the MR signal, unlike radiofrequency (RF) ablation.^2^ LITT has been applied to the percutaneous treatment of malignant hepatic and renal metastatic lesions, and primary breast and stomach carcinomas.^3^ Improvements in real-time thermal imaging with MRI, laser probe design, and computer algorithms predictive of tissue kill have led to the development of percutaneous MRI-guided laser interstitial thermal therapy (MRgLITT) and the resurgence of LITT as a treatment in neuro-oncology.^4^ Carpentier et al. first reported the safety and efficacy of LITT in metastatic brain tumors with four patients.^5^ Subsequent studies have described its use in high-grade gliomas including recurrent glioblastoma (GBM).^6,7^ Its application has increased significantly, with several case reports and case series detailing use in hypothalamic hamartomas, low-grade gliomas, refractory radiation necrosis, and hippocampal sclerosis.^1,8,9^

Several publications have described the MR appearance of LITT-treated lesions.^10–14^ However, to date, there are no published studies regarding the role of diffusion-weighted imaging (DWI) in the prediction of recurrent GBM on MRI following LITT.^10,15–19^ The current gap in knowledge is whether MRI following LITT can accurately predict the location and trajectory of GBM recurrence before the development of abnormal enhancement. An additional gap in knowledge is the immediate, mid-term and long-term appearance of post-LITT changes on DWI and ADC maps and their correlation with patient outcomes. The primary focus of this study is the correlation of diffusion changes immediately following MRgLITT. A secondary goal is to characterize the pattern and evolution of MRI changes following LITT.

Current understanding of post-LITT changes have been informed by Schober et al. and Schwabe et al.^12,20^, who describe a lesion pattern comprising five concentric zones within the first 3 months after LITT that correspond to zones of tissue injury as seen on histology, summarized in Figure 1: **(a)** The light guide track (Zone A). **(b)** The central zone of coagulative necrosis (Zone B), which contains damaged cell membranes and stains positive for markers of apoptosis. **(c)** The peripheral zone (Zone C), which contains thrombosed vessels and distended cell bodies and undergoes delayed liquefactive necrosis. **(d)** The thin rim at the outer border of the peripheral zone corresponds to the contrast-enhancing margin (Zone D) and confers a distinct “eggshell”-like appearance to the lesion due to blood–brain barrier damage. The peripheral rim gradually changes in circumference and enhancement in accordance with changes in the entire peripheral zone. It generally decreases with time or may remain stable. Residual enhancement persists on long-term follow-up, likely due to reactive inflammatory and granulation tissue. **(e)** The perifocal edema or the outermost layer of the LITT lesion is the marginal zone (Zone E).

**Fig. 1:**
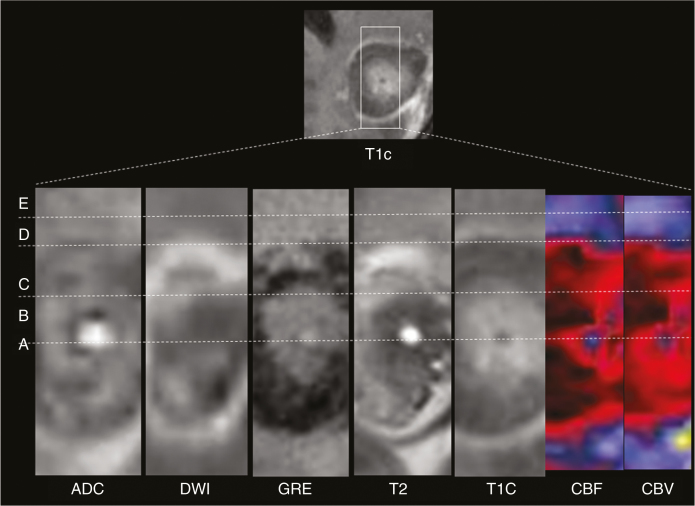
Concentric perilesional zones following LITT: ADC map, DWI, GRE, T2, T1 postcontrast (T1C), blood flow (CBF), and blood volume (CBV) images obtained 3 weeks after LITT ablation of a left parietal lesion in a 55-year-old male patient with recurrent GBM. These images demonstrate a lesion comprising five concentric zones: (**A**) probe track, (**B**) central zone, (**C**) peripheral zone, (**D**) peripherally enhancing rim, and (**E**) marginal zone (edema). Note that the concentric zones appear as inverse images on T1C and T2 images. Perfusion images (blood flow (CBF) and blood volume (CBV) demonstrate decreased blood volume in the marginal zone and in the peripherally enhancing rim.

Post-LITT lesions decrease in size exponentially after an initial expansion. The lesion enlarges to approximately 1.5 times its original size and can remain enlarged for up to 40 days due to the edema that ensues following thermal damage. Resorption of the necrotic center continues over the next few months after treatment (for at least 6 months), and during that time the associated edema diminishes and the lesion can decrease to half its original size. The enhancing periphery after contrast, similar to the shrinkage of the lesion during follow-up, shows a continuous reduction of diameter and enhancement. The residual lesion and spot-like enhancement after contrast were visible in all late controls nearly 4 years after LITT.^12,19^

However, post-LITT recurrence was not addressed in prior studies, which focused on T1- and T2-weighted images and enhancement patterns over time. There has been no analysis of post-LITT DWI and ADC changes. The basis for GBM recurrence lies in the microscopic foci of residual tumor that are known to infiltrate the peritumoral region even immediately after LITT or surgical resection.^21,22^ Given this infiltration, alterations on conventional T1-weighted contrast-enhanced MRI that are reflective of GBM recurrence are not evident for weeks to months after surgical resection, long after significant tumor infiltration has occurred.

## Materials and Methods

### Study Design and Patient Eligibility

This study was performed retrospectively by reviewing a total of 64 patients with GBM who underwent LITT at our institution between 2011 and 2017, with follow-up ranging from 6 to 70 months. All patients signed an institutional review board–approved informed consent (IRB approval: 14–1294). Ten of the 64 patients participated in a previous trial study of brain tumor treatment completed in 2013 (clinical trial registration no: NCT00747253, ClinicalTrials.gov). Of these 64 patients, 39 patients were eligible for our study as they had available DWI and ADC maps obtained on day 1 (approximately 24 h) following LITT and subsequent MRI examinations with at least 6 months of follow-up. Patients with recurrent GBM included those treated previously with resection, chemotherapy, radiation therapy, and any other additional therapies who eventually underwent LITT. Preoperative clinical data that were collected included Karnofsky Performance Status (KPS) score, previous chemotherapies, radiation therapies, and baseline neurological deficits. Participants were recruited to the brain tumor treatment study after investigator review of the individual’s clinical characteristics. Informed consent was obtained and patients were scheduled to receive the protocol treatments. All patients were not surgical candidates and had a KPS of ≥50.

### The LITT Intervention

The LITT procedures were performed using an operating room suite with an integrated 1.5-T open-bore intraoperative MRI unit. One of two primary surgeons performed the procedure using a commercial LITT system (NeuroBlate System, Monteris). This device uses a 12-watt neodymium-doped yttrium-aluminum-garnet laser emitting a 1,064 nm wavelength beam delivered to the target tissue with a carbon dioxide cooled side-firing probe.^23^ Laser catheter placement was aided with intraoperatively acquired MRI scans using a stereotactic system (Varioguide, Brainlab). Postoperatively, patients were given dexamathesone that was slowly tapered in dose. The LITT procedure was tailored for each patient based on the location of the lesion and proximity of structures such as eloquent brain, ventricles, and blood vessels. A single trajectory was used in most patients, although in some cases multiple trajectories were employed. As the goal in each case was the most complete ablation possible for each patient’s circumstances, the details of trajectory planning do not affect the analysis used in this study.

### Imaging Protocol

Standard anatomic MRI data were acquired using 1.5 Tesla MRI scanners (1.5 T: Aera or Avanto 1,100 g/cm, E11/B19 software, respectively; 1.5 T IMRIS Espree, 1,500 g/cm, B19 software; all manufactured by Siemens). Axial T1-weighted images enhanced with gadopentetate dimeglumine (Magnevist; Berlex), 0.1 mmol/kg, acquired approximately 3 min following administration of contrast material, were matched to precontrast T1-weighted images obtained with similar sequence parameters. Diffusion images were performed with conventional echo-planar (EPI) DWI (5 mm slice thickness, 1 mm slice spacing) with b-values = 0 and 1,000 s/mm^2^. A contrast-enhanced T1-weighted scan was performed on the day before the procedure for stereotactic navigation and to provide a map of the surrounding vasculature. Intraoperative imaging and ablation were performed using a head coil capable of accommodating the MR imaging calvarial bone anchor, cooling catheter, and laser probe. Intraprocedural MR thermal imaging (MRTI) provided real-time visualization of the ablation. Immediate postprocedural contrast-enhanced T1WI was performed to evaluate the effectiveness of ablation. Postoperative MRI examinations were performed within 24 h after the procedure, 1 month after the procedure and then in most cases at 2–4 month intervals thereafter, with a few patients undergoing more or less frequent follow-up. Preoperative and postoperative contrast-enhanced MR images were reviewed to assess preoperative tumor characteristics, radiographic response, and measurement of preoperative and serial postoperative enhancing tumor volumes.

### Image Analysis

Immediately after LITT, all post-LITT lesions demonstrate peripheral contrast enhancement on T1-weighted imaging (zone D). Throughout this peripheral ring, visual assessment of post-LITT patterns on DWI and ADC maps obtained 24 h following LITT were correlated with areas of GBM recurrence identified through coregistration of subsequent MRI examinations. Recurrence was identified by new abnormal tissue including increased nodular and tumefactive enhancement on T1-weighted imaging with corresponding T2 hyperintensity. Areas of suspected recurrence were confirmed by evaluation of subsequent MRI scans and correlation with clinical outcome, including death and increasing disability. Using this definition of recurrence, regions of elevated ADC in the adjacent post-LITT lesion were identified, and a specific subset of characteristic voxels was used for measurement of ADC. In these particular areas of suspected recurrence, signal intensity values on ADC maps were recorded and compared with the ADC values along the remaining peritumoral ring. Two neuroradiologists reviewed all MR examinations.

### Individual Analysis of Postoperative Apparent Diffusion Coefficient Maps 24 h Following LITT

Raw image data from apparent diffusion coefficient (ADC) and DWI acquisitions were aligned to the volumetric T1-weighted contrast-enhanced sequence with the use of the FMRIB (Oxford Centre for Functional MRI of the Brain) linear image registration tool.^24^ An affine coregistration scheme (linear transformation involving rotation, translation, and scaling is employed for alignment of different MRI protocols, as well as for pre-, and post-LITT MRI at different time-points for evaluating LITT response.^24^ Calculated ADC maps (with values multiplied by 10^–6^ mm^2^/s) were used directly for image analysis.

### Definition of Responders

Patients with a decrease in the volume of contrast-enhancing tumor in each MRI session from the first postoperative study to 6 months postprocedure and who had no evidence of clinical progression were designated “responders.” For recurrent GBM cases, the National Institutes of Health (NIH) Recurrent GBM Scale score was calculated, with 1 point each for KPS score of ≤ 80, tumor size ≥ 50 mL, and involvement of two or more eloquent regions of the brain.

### Statistical Analysis

Statistical analysis was performed using MATLAB (MathWorks). Within the peritumoral region, areas of future GBM recurrence were identified through coregistration of follow-up MRI examinations and compared to signal intensity on ADC maps obtained on day 1 following LITT. Comparison was performed using Wilcoxon rank sum tests. Optimal threshold values for ADC were obtained by area under the curve (AUC) analysis derived from the receiver operating characteristic (ROC) curves and maximizing the sum of sensitivity and specificity. Linear regression analysis was performed with *P* < .05 considered statistically significant. Multivariate analysis was not performed due to the small number of events. The significance of individual regression coefficients was tested using the Wald statistical t test. ROC curves were generated to assess the accuracy of binary classification of recurrent and nonrecurrent areas on the basis of signal intensity.

## Results

Out of the 39 eligible patients, 36 patients had proven radiologic recurrence after LITT and 3 were LITT “responders.” The characteristics of patients with recurrent GBM after LITT and LITT responders are summarized in Table 1.

**Table 1:** Data and outcomes pre- and post-LITT for 39 patients with GBM

Characteristics	Recurrent GBM after LITT	LITT Responder
Number of patients (39 total)	36	3
Mean age in years	51.7	68 (55, 74, 75)
Gender	19M, 17F	2M, 1F
Median months from diagnosis	25	19
Location of primary lesion	13 Parietal, 10 frontal, 5 temporal, 2 occipital, 2 corpus callosum, 1 cingulate, 3 thalamic	Left parietal; deep left parietal; left frontal
Lesion in eloquent area	21	1
Post-LITT recurrence location		
Marginal/peritumoral:	26	None
Within tumor:	4	
Distant multifocal:	6	
Treatment prior to LITT	17	1
Surgical total resection:	15	1
Surgical subtotal resection:	4	1
Biopsy only:	7	
Stereotactic radiation therapy:	29	
Fractionated radiation therapy:	6	
Bevacizumab:	30	
Temozolomide:		
Patients with neuro deficit	27	1
Mean pre-LITT tumor volume (cm^3^)	15.4	9.2
Avg NIH Recurrent GBM Scale score	1.1	1.3
Median % change in volume 3 months post-LITT	62%	−56%
Median PFS	6 months	NA*
Median survival after LITT	>7 months	NA*

Avg, average; NA, not applicable; neuro, neurological; PFS, progression-free survival.

*Two patients still alive and are still progression free.

In the peritumoral areas of suspected recurrence, analysis of signal intensity values on ADC maps permitted sensitive detection of signal changes, despite the marked signal heterogeneity that is typical in the peritumoral region following LITT. The mean ADC value within areas of subsequent recurrence was 1,391.3 × 10^–6^ mm^2^/s compared with 1,012.7 × 10^–6^ mm^2^/s within the surrounding peritumoral region, representing an overall 37% increase in the ADC value (*P* < .01). On the basis of the Wald statistic, the signal intensity values on ADC maps are a statistically significant predictor of tumor recurrence (*P* < .01).

### Group with Recurrent GBM

Thirty-six subjects had recurrent GBM after LITT. Gender, age, and treatments prior to LITT are illustrated in Table 1. The median KPS score was 80. The LITT procedure was performed a median of 25 months from initial diagnosis. At the time of the LITT procedure, most subjects with subsequent recurrence had intermediate NIH Recurrent GBM Scale scores: 6 patients had a score of 0, 21 had a score of 1, and 9 had a score of 2. The mean preoperative enhancing tumor volume was 15.4 mL. Recurrence was identified as new, expansile, abnormally enhancing tissue on follow-up MRI scans. The median time to radiographic or clinical progression was 6 months, and the median overall survival was greater than 7 months from the LITT procedure; 15 patients were alive at the time this study was performed.

**Fig. 2:**
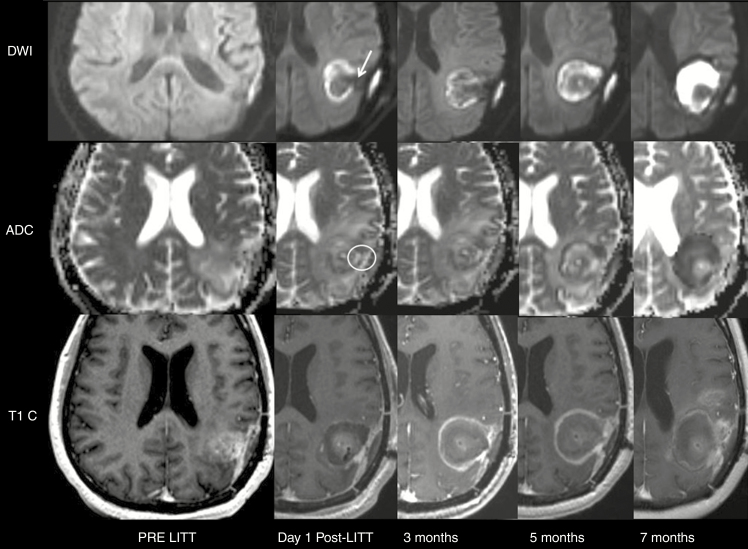
Typical MRI scans before and after LITT in 43-year-old male with GBM with recurrence after LITT: On day 1 post-LITT, there is a discontinuity in the peripheral restricted diffusion (white arrow) with corresponding decreased DWI signal and increased ADC (white circle) within the expected periphery of restricted diffusion 24 h post-LITT. This corresponds to an area of new peripheral enhancement on T1 postcontrast images obtained after 5 months, consistent with recurrence. The site of discontinuity on DWI obtained at day one after LITT predicts the location of recurrence in the subsequent scans in this case.

Of the 36 subjects with proven radiologic recurrence after LITT, 31 had areas of decreased DWI signal and increased signal on ADC maps within adjacent sections of the expected peritumoral ring of restricted diffusion on scans performed within 24 h following LITT that corresponded to sites of GBM recurrence on subsequent MRI examinations (*P* < .01), detected with a sensitivity and specificity of 86.1% and 75.2%, respectively. A significantly greater area under the receiver operating characteristics curve was determined for ADC values (*P* < .01). These results suggest that location of GBM recurrence after LITT can be predicted as a function of signal intensity on ADC maps and DWI images (Figure 2).

### Group of Responders

**Fig. 3:**
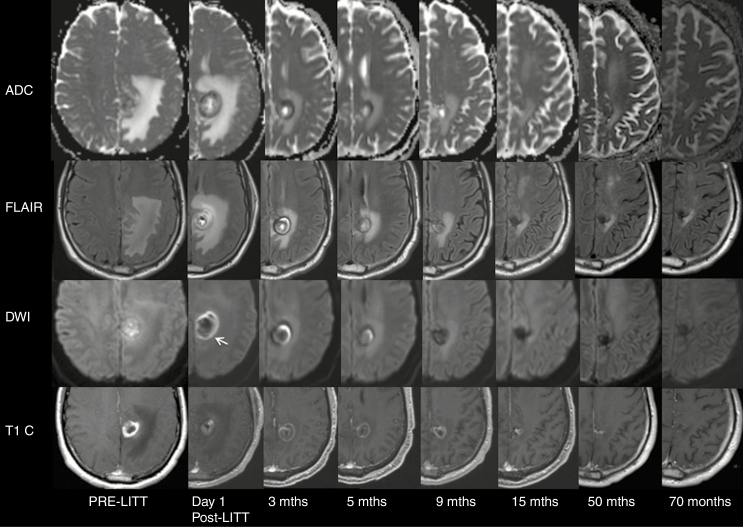
MR imaging (ADC maps, T2 FLAIR, DWI, and T1 postcontrast images) in a representative case without recurrence of a left parietal GBM treated with LITT. At day 1 post-LITT, there is a complete undisrupted periphery of decreased ADC and DWI hyperintensity (white arrow) at the edge of the treatment zone that marks the border of the peripheral rim of enhancement (representative border shown, not all images are included in this figure). The peripheral restricted diffusion shrinks exponentially parallel to the peripheral enhancement and was resolved after 9 months. Contrast enhancement decreased gradually in the first 15 months, stable up to 5 years and resolved after 70 months.

Of the 39 total subjects in this study, 3 subjects were classified as responders, with durably decreased enhancing tumor volume following ablation. At the time of the LITT procedure, two subjects had an NIH Recurrent GBM Scale score of 1 and one subject had a score of 2. Compared with the recurrent GBM group, the average preoperative tumor volume was smaller (9.2 mL). The median KPS score was similar (80). Among the three responders, two were still alive at the time that this study was initiated. Figure 3 shows preoperative, 24-h post-LITT, and 70-month follow-up MR images of a “responder” subject who underwent LITT for recurrence of GBM in the left parietal lobe.

## Discussion

In subjects with GBM treated with surgical resection or LITT, signal abnormality in the peritumoral region represents a complex conglomerate of edema, gliosis, and residual tumor.^25^ Differentiation between these separate entities through visual inspection of conventional MRI is very difficult. As a result, imaging evaluation of residual disease in patients with treated GBM is challenging.^26,27^

Within the peritumoral region, there have been mixed results in extracting prognostic information from differences in diffusivity after surgical resection of GBM,^28–32^ but no such studies have been published regarding diffusion imaging following LITT. Evidence suggests that, following surgical resection, microscopic GBM infiltration may induce slight variations in the ADC and the signal intensity within the peritumoral region on T2-weighted images, including T2 FLAIR images. Early studies have shown that small increases in restricted diffusion correlate with increased tumor cellular density.^33,34^ Recently published findings have also shown decreased ADC in the peritumoral region of infiltrative tumors such as GBM, in comparison with less invasive brain metastases.^28,33^ However, these findings were observed following surgical resection of GBM, and have not yet been reported following LITT. In 2017, Chang et al. studied MRI scans from 26 patients with GBM immediately following tumor resection. These investigators found that areas of future GBM recurrence exhibit small but highly statistically significant differences in signal intensity on ADC maps and T2 FLAIR images. Sites of subsequent tumor recurrence showed a 9.5% decrease in the ADC value (*P* < .01) and a 9.2% decrease in T2 FLAIR signal intensity (*P* < .01), compared with adjacent parenchyma with peritumoral edema and gliosis.^33^ However, to date, the literature that has investigated GBM recurrence based on MRI following LITT^10,15–19^ has not addressed diffusion changes, and so, these changes remain uncharacterized.

In this study, we identified a new finding correlated with recurrence of GBM: On scans performed within 24 h following LITT, all 39 GBM lesions showed a periphery of restricted diffusion at the edge of the treatment zone that marks the inner border of the peripheral rim of enhancement (zone D) and corresponds to the outer edge of liquefactive necrosis. The periphery of restricted diffusion shrinks exponentially parallel to the enhancing ring and resolves after 9 months. A complete undisrupted periphery of restricted diffusion on a scan performed 24 h following LITT that shrinks exponentially on subsequent examinations signifies a favorable prognosis (*P* < .01; Figure 3). In contrast, the presence of a discontinuity in this peripheral restricted diffusion, identified by decreased DWI signal and increased signal on ADC maps, identified on MRI scans obtained 24 h following LITT correlated with sites of subsequent GBM recurrence (Figure 2). We hypothesize that these areas of recurrence are insufficiently treated and therefore do not have necrosis like the remainder of the ring of restricted diffusion. Therefore, these may reflect regions that are either lacking tumor death rather than the tumor itself or may harbor residual infiltrative GBM. An alternative explanation, rather than the cellular infiltration and/or zone of incompletely treated tumor as early as 24 h after LITT, could be related to a zone of reduced resistance to infiltration based on the presence of a matrix or a mechanical breach in the matrix along the laser probe trajectory that is more permissive of, or perhaps, even promotes invasion. These findings suggest that the location of GBM recurrence following LITT may be predicted in a scan obtained within 24 h of LITT. This information might alter treatment planning, perhaps suggesting areas that may be targeted for additional therapy.

Multiple studies have demonstrated that recurrence following LITT tends to occur within the peripheral rim of enhancement and presents as new or enlarging enhancing nodularity or simply as thickening of the peripheral enhancement.^7,35^ Comparison with prior images is vital in monitoring tumor recurrence because post-LITT changes can include asymmetric enhancement similar to that of recurring lesions. Therefore, irregularly enhancing lesions require close follow-up. Any increase in size, enhancement, or surrounding edema should be further assessed with adjunctive techniques such as MR spectroscopy, perfusion imaging, or PET.^36^

A strength of our study is that it provides a larger number of patients and longer period of follow-up than any currently reported study of LITT. Some limitations of this study include its retrospective design and consequent problems of selection bias. A subset of patients was treated with radiation therapy and concurrent temozolomide as part of a clinical trial and received additional trial drugs that may have influenced tumor cell behavior, possibly including the pattern of disease progression after LITT. This can also be affected by the mutational status of patients such as IDH1 and MGMT methylation status which were not provided in the entire population of our study. Another limitation is the complexity of MRI signal changes within the central and peripheral zones, particularly at day 1 after LITT due to the presence of hemorrhage, which may affect signal characteristics; however, our measurements are within the peritumoral region, which is least affected by signal heterogeneity and should correspond to areas of GBM progression and recurrence. Additional limitations are the lack of site-specific neuropathologic correlation to definitively distinguish between tumor recurrence and pseudoprogression, however, we believe that the definition of responders compared to recurrence that we used in our model based on follow-up scans and patient’s clinical decline was critical to minimize this issue. A potential confounder is the interscanner variation in ADC values, which was reduced but not eliminated in our study since all the MRI scanners were manufactured by the same vendor and used similar magnetic field strength and coil systems. Moreover, we have attempted to alleviate the effects of interscanner variability by calculating the ratio of the ADC value in the gap to the remainder of the expected peritumoral ring of restricted diffusion in each patient. However, of the 36 subjects with proven radiologic recurrence after LITT, this gap of increased ADC signal within the peritumoral ring of restricted diffusion was visually perceptible in 31 patients, so its detection would be practical in the clinical setting, without the need for the calculation of actual values. Therefore, the practical point might be made that the ADC gap can be identified based on visual analysis. The imaging used for analysis and measurement of ADC values is relatively low resolution, which limits the accuracy of the data used in this study. Higher-resolution diffusion imaging would be useful to confirm the findings of this study.

In summary, LITT is an emerging minimally invasive treatment modality that is increasingly used in the treatment of diverse brain lesions. However, there has been limited research regarding prognostic features related to tumor progression and recurrence in patients with GBM following LITT. The findings of our study suggest that within the complex conglomerate of signal abnormality seen in patients with GBM after LITT, areas of decreased DWI signal and increased ADC along the expected peritumoral restricted diffusion on scans performed 24 h after LITT are likely to harbor residual tumor that may be sites of future recurrence. Further prospective investigation with multiparametric logistic regression models would be helpful to better characterize imaging changes, perhaps using quantitative modeling techniques to analyze variations on a voxel-by-voxel basis to discern the distribution of otherwise imperceptible tumor infiltration that may contribute to progression and recurrence. If successful, this may result in a novel approach for both prediction and treatment of GBM recurrence. While these results are derived from analysis of treated brain lesions, we speculate that a similar phenomenon may apply to LITT in other body tissues.

## Notes

**Authorship statement.** Study conception and design: A.M., M.L., G.B., A.M.M. Acquisition of data: A.M., M.L., G.B., A.M.M. Analysis and interpretation of data: A.M., M.L., S.B., E.E., G.B., A.M.M. Drafting of manuscript: A.M., M.L., S.B., E.E., A.M.M., G.B. Critical revision: A.M., M.L., S.B., E.E., A.M.M., G.B.
